# Theory, models and biology

**DOI:** 10.7554/eLife.07158

**Published:** 2015-07-14

**Authors:** Wenying Shou, Carl T Bergstrom, Arup K Chakraborty, Frances K Skinner

**Affiliations:** Division of Basic Sciences, Fred Hutchinson Cancer Research Center, Seattle, United Stateswenying.shou@gmail.com; Department of Biology, University of Washington, Seattle, United Statescbergst@u.washington.edu; Departments of Chemical Engineering, Physics, Chemistry and Biological Engineering, and the Institute for Medical Engineering & Science, Massachusetts Institute of Technology, Cambridge, United Statesarupc@mit.edu; Toronto Western Research Institute, University Health Network and the University of Toronto, Toronto, Canadafrances.skinner@gmail.com

**Keywords:** research, theoretical biology, mathematical modelling, computational biology, evolution, scientific publishing

## Abstract

Theoretical ideas have a rich history in many areas of biology, and new theories and mathematical models have much to offer in the future.

When scientists want to explain some aspect of nature, they tend to make observations of the natural world or collect experimental data, and then extract regularities or patterns from these observations and data, possibly using some form of statistical analysis. Characterizing these regularities or patterns can help scientists to generate new hypotheses, but statistical correlations on their own do not constitute understanding. Rather, it is when a mechanistic explanation of the regularities or patterns is developed from underlying principles, while relying on as few assumptions as possible, that a theory is born. A scientific theory thus provides a unifying framework that can explain a large class of empirical data. A scientific theory is also capable of making predictions that can be tested experimentally. Moreover, a theory can be refined in the light of new experimental data, and then be used to make new predictions, which can also be tested: over time this cycle of prediction, testing and refinement should result in a more robust and quantitative theory. Thus, the union of empirical and quantitative theoretical work should be a hallmark of any scientific discipline.

Theory has long been celebrated in the physical sciences, but the situation is very different in the life sciences. As Conrad Hal Waddington wrote in 1968, in the preface of *Towards a Theoretical Biology*: ‘Theoretical Physics is a well-recognized discipline, and there are Departments and Professorships devoted to the subject in many Universities. In strong contrast to this situation, Theoretical Biology can hardly be said to exist as yet as an academic discipline. There is even little agreement as to what topics it should deal with or in what manner it should proceed’.

Yet theory plays a paramount role in biology. The best known example of a theory in biology is, of course, the theory of evolution by natural selection. Charles Darwin may have been a globe-trotting hands-on naturalist and geologist, but his outstanding contribution to science was theoretical. Drawing on fieldwork, fossil records and the breeding records of domestic animals and plants, he observed that variations readily arose and that much of this variability was heritable. After reading Malthus' essay on the repercussions of an exponential growth in population, Darwin reasoned that a struggle for existence must have selected for the variants that were most adapted to their local environment. As different populations adapted to different environments, he argued that these variations accumulated over time, eventually forming diverse species. Despite the success of his theory, Darwin never formalized it in mathematical terms. Rather, he wrote: ‘I have deeply regretted that I did not proceed far enough at least to understand something of the great leading principles of mathematics; for men thus endowed seem to have an extra sense’ (May, 2004). Although a theory does not have to be formulated as a mathematical model to be useful, the development of such a model is a hallmark of a maturing theory. The role of theory and mathematical models in the life sciences is the focus of this editorial.

The best known example of a theory in biology is, of course, the theory of evolution by natural selection.

By the end of the 1960s, when Waddington was bemoaning the lowly status of theoretical biology, the field had in fact witnessed major breakthroughs. Early population geneticists such as Pearson, Fisher, Wright and Haldane had developed the formulation that Darwin was unable to construct, providing a mathematical foundation for the theory of evolution by natural selection. In the process, they also generated a number of major advances in statistics. The modern evolutionary synthesis had reconciled the gradualist Darwinian view of natural selection with a Mendelian understanding of genetics, unifying observations from naturalists, experimental geneticists and paleontologists. A crucial contribution from theory came in 1943 when Luria and Delbrück used mathematical reasoning and experiments to conclude that mutations arose in the absence of selection, rather than in response to selection. And in 1953 the structure of DNA was determined with the help of a theoretical physicist and the building of physical models (which were the forerunners of today's computer simulations). Elsewhere, the simple and elegant Lotka–Volterra models of competition and prey-predation had jump-started theoretical ecology, Kermack–McKendrick theory had laid a foundation for mathematical epidemiology, and Burnet had developed the clonal selection theory that lies at the heart of our understanding of the adaptive immune system. In neuroscience it is difficult to overstate the importance of the Hodgkin–Huxley model of action potentials or Rall's use of cable theory to provide a framework for understanding the complex, dendritic structures of neurons.

During the past half-century, theory has continued to advance in diverse areas of biology. Within evolution and ecology, for example, evolutionary game theory provided a framework for thinking about the evolution of strategic behavior, while kin selection and multi-level selection theory helped to explain cooperation and altruism. Life history theory offered a systematic way to think about the evolution of senescence, developmental plasticity and reproductive schedules, among other things, while optimal foraging theory introduced economic reasoning into the study of animal foraging. Other examples include kinetic proofreading in biochemistry, the Hopfield model of neural networks, and the use of bifurcation theory and phase-plane analyses in neuroscience.

Increased computational power has also allowed biologists to study the structure and function of proteins, and to simulate complex biological processes such as morphogenesis, chemotaxis, the cell division cycle, metabolism and, in some cases, the workings of the entire cell. And over the past decade new experimental tools and techniques have generated such a staggering amount of data that we are, in the words of Sydney Brenner, ‘thirsting for some theoretical framework with which to understand it’ (Brenner, 2012). This is true in genetics and genomics, immunology, microbiology, neuroscience and many other areas. New theoretical and computational models are therefore needed to make sense of this abundance of data.

Yet, despite this rich history, the divide between theoretical and empirical biologists seems to persist, even in areas with a long history of both types of work, such as ecology and evolutionary biology (Haller, 2014). One reason for this is that the complexity of real biological systems often requires relatively sophisticated mathematics, which means that many theoretical papers do not resonate with empirical biologists. This complexity has many sources: the number of interacting parts in even the simplest living cell presents a formidable challenge for a theoretical biologist, as does the heterogeneity that is intrinsic to biological systems. Moreover, interactions among these parts can span a large range of time scales (from picoseconds for electron transfer in photochemical reactions, to billions of years for evolution) and length scales (from molecules to cells, from organisms to ecosystems).

Yet, despite this rich history, the divide between theoretical and empirical biologists seems to persist, even in areas with a long history of both types of work, such as ecology and evolutionary biology.

As a result, theoretical biologists often need to make a trade-off between abstraction and realism (or between the qualitative and the quantitative) when building mathematical models. The appropriate level of abstraction will depend on the question of interest. For example, simplifying assumptions can be made to develop a highly abstract model that reveals general features shared by many systems and thus improves our understanding of some aspect of biology. However, such a model is unlikely to produce quantitative predictions for any particular system. On the other hand, a highly detailed model that contains many equations and parameters is unlikely to improve intuitive understanding of a system or process. However, if the various parameters in the model can be measured to a credible level, then these models should be able to make quantitative predictions about a given system or process. Part of the challenge in model building is to choose the right level of abstraction despite the complexity of biological processes. In other words, we need to work out what aspects of this biological complexity we can ignore and still gain critical insights about a biological phenomenon.

So how can we increase interactions and collaborations between theoretical biologists and empirical biologists for the benefit of the discipline as a whole? First, universities and institutions should ensure that biology students are taught more about theoretical and mathematical techniques, including ideas from physics that have already been successfully applied to biological questions (such as statistical mechanics and nonlinear dynamics). Laboratory work could also be extended to include exercises that involve computer simulations. These changes would help biologists to better communicate with theorists and, more importantly, to incorporate quantitative thinking into their own work. There are signs that this is starting to happen: the sixth edition of *Molecular Biology of the Cell*, for example, includes examples where ordinary differential equations are used to model gene regulation and to explain switch-like and oscillatory dynamics. It would be good to see more mathematics in biology textbooks.

Second, theoretical biologists could do more to increase the chances that their papers will resonate with empirical biologists. The primary audience for some theory papers will be other theorists, and like all papers aimed at a specialist readership, these papers will be a challenging read for non-specialists. However, the potential impact of most theoretical papers—especially modeling papers—could be increased by following a few simple guidelines. The first thing to do is to clearly state the goal of the modeling: is the aim to organize data emerging from high-throughput experiments, to test a particular hypothesis, to uncover the basic mechanisms driving some phenomenon, to evaluate the feasibility of an intuitive argument, to make specific predictions, or something else? How does the model or theory relate to and differ from previous models, and what are its advantages and disadvantages? What assumptions have been made, and what are the justifications for these? How were the parameters in the model chosen?

Theoretical biologists could do more to increase the chances that their papers will resonate with empirical biologists.

Mathematical papers can be made more accessible by giving step-by-step derivations for equations, and intuitive explanations for how these equations reflect the biological process under investigation, even if this involves covering material that may already be familiar to other theoretical biologists. Schematic diagrams can also help. Finally, it is important to relate the conclusions back to biology. This includes clearly stating which conclusions are not surprising (in the sense that they are straightforward derivatives of the empirical results used to constrain a model), which insights are novel, and which predictions are worthy of empirical tests. Theoretical biologists can also benefit from wet lab experience to help them appreciate what doing an experiment involves.

Third, empirical biologists could make their work more accessible and valuable to theorists. For example, all the relevant datasets should be included in papers. Moreover, where possible, time-course data should be collected, rather than just ‘end-point’ data, as this will allow dynamical processes to be studied. And when the experimental measurements in a paper differ from previous measurements in a significant way, it would help theorists (and others) to build on the work if the authors discussed possible reasons for these differences. Taken together the recommendations outlined above should lead to improved collaboration between theoretical and empirical biologists.

*eLife* welcomes theoretical and modeling papers in all areas of biology, especially papers that report new biological insights, make substantial predictions that can be tested, or help to resolve contradictory empirical findings. Papers that report new theories or algorithms that have the potential to solve important biological problems are also welcome. Papers can also be as long (or as short) as necessary. Across the life sciences we aim to publish papers that are insightful and change the way that other researchers think about their subject (Malhotra and Marder, 2015). Theory and modeling are no exception.

## References

[bib1] Brenner S (2012). Life's code script. Nature.

[bib2] Haller BC (2014). Theoretical and empirical perspectives in ecology and evolution: a survey. BioScience.

[bib3] Malhotra V, Marder E (2015). The pleasure of publishing. eLife.

[bib4] May RM (2004). Uses and abuses of mathematics in biology. Science.

